# Digestive Enzymes of the Crustaceans *Munida* and Their Application in Cheese Manufacturing: A Review

**DOI:** 10.3390/md9071220

**Published:** 2011-07-07

**Authors:** Rocco Rossano, Marilena Larocca, Paolo Riccio

**Affiliations:** Department of Biology, Defence and Agro-Forestal Biotechnology, Center of Bioproteomics, University of Basilicata, 85100 Potenza, Italy; E-Mails: laroccamarilena@libero.it (M.L.); paolo.riccio@unibas.it (P.R.)

**Keywords:** crustaceans, enzymes, proteases, cheese

## Abstract

Crustaceans *Munida* (fam. *Galatheideae*, ord. *Decapodi*) were fished in the Southern Adriatic Sea and their proteolytic activities were characterized and tested for potential application in cheese manufacturing. Enzymes extracted from whole crustaceans, mainly serine proteases, showed high caseinolytic and moderate clotting activities. Analysis by 2D zymography of the digestive enzymes extracted from *Munida* hepatopancreas, showed the presence of several isotrypsin- and isochymotrypsin-like enzymes in the range of 20–34 kDa and 4.1–5.8 pI. Moreover, specific enzymatic assays showed the presence of aminopeptidases and carboxypeptidases A and B. Overall, optimum activity was achieved at pH 7.5 and 40–45 °C. Caseinolytic activity, determined both spectrophotometrically and by SDS gel electrophoresis, indicated higher activity on β-casein than on α-casein. Miniature cheddar-type cheeses and Pecorino-type cheeses were manufactured by adding starter, rennet and *Munida* extracts to milk. Reverse-phase HPLC and MALDI-ToF mass spectrometry showed a more complex pattern of proteolytic products in cheeses made using *Munida* instead of chymosin. *Munida* extracts were found to degrade the chymosin-derived β-casein fragment f193–209, one of the peptides associated with bitterness in cheese. In conclusion, *Munida* digestive enzymes represent a promising tool for development of new cheese products and shorten cheese ripening when used either alone or in addition to calf rennet.

## 1. The Interest for Marine Enzymes in Biotechnological Processes

Marine enzymes represent a special class of biocatalysts since they are inside organisms living in an environment characterized by high pressure, high salinity, low temperature, little sunlight, *i.e.*, in conditions which are very different from those of the terrestrial environment. On these grounds, marine enzymes might have particular physical, chemical, and catalytic properties that could be of advantage in several biotechnological processes [1].

Therefore, marine enzymes have been suggested for many industrial applications such as pharmaceuticals, cosmetics, nutritional supplements, molecular probes, food additives, fine chemicals, and agrichemicals [2–5]. In particular, the use of sea-derived enzymes in food technology is becoming a promising application for the development of new processes and new products, including substitution of rennet in cheese manufacture; removal of the oxidized flavor from milk; ripening and fermentation of fish products; and preparation of fish protein hydrolysates and concentrates [6–9].

Proteolytic enzymes have important applications in the food industry [10]. In particular, there is a growing interest for digestive proteases from marine sources due to their activity at low temperature and the availability of raw materials such as viscera for their extraction. Fish viscera are indeed a rich source of digestive enzymes, such as pepsin and the serine proteases, trypsin and chymotrypsin [11,12]. The property of the digestive enzymes from marine organisms to maintain their activity at low temperature might be very useful in food processing in order to avoid bacterial contamination and unwanted chemical reactions [13]. Serine proteases such as elastase and collagenase are digestive enzymes found in fishes and marine invertebrates such as crab, prawn and lobster [7,10,11]. There is a particular interest in studying proteolytic enzymes from marine species both as possible substitutes for milk-clotting enzymes and for the shortening of cheese ripening times. These potential applications are based on the finding that some gastric proteases from marine species may have chymosin-like properties [14–18]. But, most interesting in the present context, is the finding that some gastric proteases from marine species have chymosin-like properties [14–18] and therefore may substitute chymosin in milk-clotting activities and shorten cheese ripening times.

## 2. The Crustaceans *Munida* as a Source of Digestive Enzymes for Cheese Technology

**Figure 1:**
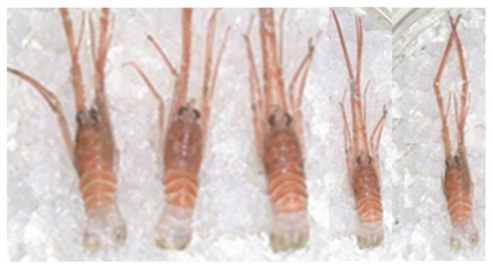
*Munida* crustaceans.

With regard to the application of marine enzyme in the manufacture of cheese, we have introduced the use of the crustaceans *Munida* (fam. *Galatheideae*, ord. *Decapodi*) (Figure 1) as a possible source of milk coagulating enzymes and as adjutants in cheese ripening, and we have described and characterized their proteolytic pattern, as well as their ability to make cheese.

*Munida* crustaceans live mainly in the Atlantic Ocean and deep Mediterranean bottoms [19]. They are not vulnerable, endangered or protected and have no commercial value, but they are occasionally caught and discarded at sea. Most of the specimens die during fishing targeted to other commercial fish, thus most of the discard is not alive. Finding another potential use for these crustaceans could help to augment the revenue of fishermen.

In our studies, the *Munida* crustaceans were fished in the Southern Adriatic Sea, near the town of Bari (Italy). The crustaceans were identified and controlled at the Institute of Marine Biology of Bari, transported on ice to our laboratory and stored at −70 °C until use. The significant presence and diversity of proteases in these marine organisms can be attributed to the fact that these shellfish depend on their diet to provide essential amino acids and must therefore have an efficient digestive system.

Highly active proteolytic enzymes acting on alimentary proteins have been reported in many marine species [20–22]. Proteolytic enzymes in the digestive organs of crustaceans have been well documented and characterized [18,23–29]. Marine decapod crustaceans synthesize a wide range of highly active proteolytic enzymes in the digestive gland: endopeptidases (trypsin and chymotrypsin) and exopeptidases (carboxypeptidases and aminopeptidases).

## 3. The Enzymes of Whole *Munida* Crustaceans

In the first experiments, enzymatic activity was extracted by breaking and homogenizing whole crustaceans (*n* = 15) with deionized water. After centrifugation, the proteolytic activity present in the supernatant was determined by measuring the degradation of azocasein in solution and characterized by zymography.

The extract showed a optimum of proteolytic activity at pH 6.5–7.5 and at a temperature of 55–60 °C [30]. Activity was quite stable. After 40 days at −20 °C, 75% of the initial proteolytic activity was still present. Studies carried out with specific substrates and inhibitors, showed the presence of several proteolytic enzymes, mainly serine proteinases such as trypsin and chymotrypsin.

The premise for the application of *Munida* enzymes in cheese production was to determine their ability to degrade the caseins and to check for the presence of milk-clotting activity. The extracts showed high proteolytic activity on caseins, and moderate coagulant activity, determined according to the FIL-IDF 157/92 norm. In particular, the coagulant activity of the extracts obtained from the crustaceans was 150 times lower than the traditional commercial liquid calf rennet of Clerici (Caglio Liquido, Caglificio Clerici, Cadorago, Italy), and 80 times lower than the common lamb rennet pastes [30].

## 4. The Digestive Enzymes from the Hepatopancreas of Crustaceans *Munida*

On the basis of preliminary data obtained from the enzyme extracts of whole crustaceans indicating high caseinolytic capability and moderate clotting activity [31], subsequent studies were conducted using the enzymes extracted from the hepatopancreas of the *Munida* crustaceans, in order to obtain extracts enriched in digestive enzymes only. For the extraction of the digestive enzymes from *Munida*, their hepatopancreas was collected from 25 individuals and homogenized in 10 mM sodium phosphate buffer, pH 7.0, and 100 mM NaCl. The supernatant obtained after centrifugation was passed through 0.45 μm filters and used for the characterization of the extracted digestive enzymes. Proteolytic activity was determined by measuring azocasein breakdown in solution. The extracts showed optimum activity at pH 7.5 and 40–45 °C, respectively. As determined by the use of specific inhibitors, most of the enzymes were found to be serine proteinases, but some metalloproteinases were also present. Further characterization of proteolytic and peptidase activities present in the hepatopancreas extracts was performed using specific enzymatic assays. The extracted enzymes were able to hydrolyze the substrates benzoyl-Arg-*p*-nitroanilide (BAPNA) and *N*-succinyl-Ala-Ala-*pro*-Phe-*p*-nitroanilide (SAPNA), indicating the presence of trypsin-like and chymotrypsin-like activities, respectively. Analysis of peptidase activities present in the *Munida* extracts showed the presence of carboxypeptidases A and B, and the presence of several aminopeptidases. Among them, the following amminopeptidases were detected: PepN and PepC, aminopeptidases with broad specificity; PepA, aminopeptidase specific for Glu/Asp residues; PepI, iminopeptidase capable of releasing an *N*-terminal proline residue; and PepX or prolyl-dipeptidil aminopeptidase, a proline-specific peptidase [31].

To assess the caseinolytic activity, the extracts obtained from the hepatopancreas of the crustaceans *Munida* were incubated with casein. Analysis of the digested products, performed both spectrophotometrically and by SDS gel electrophoresis, indicated that enzymes extracted from the hepatopancreas of *Munida* have a higher activity on β-casein than on α-casein.

**Figure 2:**
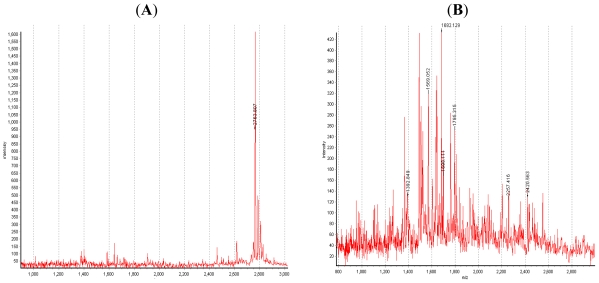

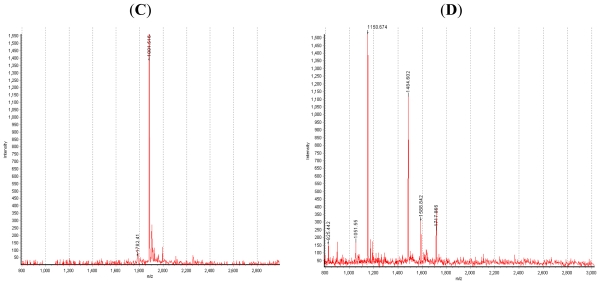
Peptide profile performed by MALDI-ToF mass spectrometry analysis. MALDI ToF mass spectra of peptide mixture resulting from the activity of: (**A**) commercial rennet Clerici on α-casein; (**B**) enzymes extracted from the hepatopancreas of the crustaceans *Munida* on α-casein; (**C**) commercial rennet Clerici on β-casein; (**D**) enzymes extracted from the hepatopancreas of the crustaceans *Munida* on β-casein.

Through analysis of the peptide profile, performed by Matrix Assisted Laser Desorption Ionisation Time of Flight (MALDI ToF) mass spectrometry, we compared the activity of digestive enzymes of the Munida with that of the commercial liquid calf rennet Clerici on the fractions of α- and β-casein. The results obtained (Figure 2) were clearly different.

In particular, the α-casein peptide profile resulting from the activity of commercial rennet Clerici (spectrum A), showed only the peptide of 2763.8 Da, corresponding to the fragment α_S1_-casein (f1-23) deriving from the hydrolytic activity of chymosin on Phe_23_–Phe_24_ bond [32,33], while the peptide profile (spectrum B) corresponding to the action of *Munida* enzymes is very complex and is characterized by the presence of at least 20 peptides. With regard to the activity on β-casein of the commercial rennet Clerici, the peptide profile showed only one peak with molecular mass 1881.5 Da (spectrum C), corresponding to the peptide β-CN (f193–209), due to the activity of chymosin on β-casein at Leu_192_-Tyr_193_ [34]. This peptide, widely reported as the major cause of bitterness in cheeses [35–37], was not found in the peptide mixture obtained after hydrolytic treatment of β-casein with the digestive enzymes of *Munida* (spectrum D), suggesting that *Munida* enzymes might play an important role in the degradation of bitter peptides in cheese. Studies performed by MALDI-ToF mass spectrometry on the degradation of the bitter peptide β-CN (f193–209) by the *Munida* enzymes [38] showed that *Munida* enzymes are able to degrade the chymosin-derived β-casein fragment f193–209 [31]. Peptides deriving from its degradation might be the result of aminopeptidase activity [38,39].

## 5. Detection of *Munida* Proteolytic Activities by Casein Gel Zymography

Analysis by monodimensional zymography was undertaken to determine the composition and the molecular mass of the digestive enzymes present in the *Munida* hepatopancreas. Casein was chosen as a substrate. 11 activated digestion bands were detected in the range of 76–18 kDa. Most of them were serine proteinases. This finding was consistent with the results reported for other crustaceans by other authors [40,41].

To identify the *Munida* proteome corresponding to the proteolytic activities in more detail*,* the extracts were applied to 2D casein gel zymography, a technique that allows the specific determination of both molecular masses and isoelectric points of proteases in a complex protein mixture. Samples were prepared for the first dimension separation (IEF) in the absence of DTT in order to retain the enzymatic activity of the caseinolytic enzymes. Proteolytic enzymes were separated and detected after the second dimension SDS gel casein zymography by staining with Coomassie blue. The resulting zymograms showed the presence of 12 spots (clear unstained zones) indicating the presence of proteolytic enzymes in the range of 20–34 kDa and 4.1 to 5.8 pI. Results showed the presence of several isotrypsin-like and isochymotrypsin-like enzymes. In particular, six different acidic forms of trypsin were detected using specific inhibitors, trypsin-like activity was higher than chymotrypsin-like activity [31]. Apparent molecular masses and isoelectric points were similar to those of digestive enzymes from other crustaceans [22,42–44].

## 6. Cheesemaking Trials Using the Enzymes Extracted from the Hepatopancreas of *Munida* Crustaceans

Two different types of cheeses were manufactured with the digestive enzymes extracted from the hepatopancreas of *Munida*.

### 6.1. Mini Cheddar-Type Cheeses

The well-established model of miniature Cheddar-type cheeses was used to investigate whether the extracts from the hepatopancreas of *Munida* are suitable for cheese making. In a study carried out in collaboration with the University College of Cork, Ireland, miniature (20 g) Cheddar-type cheeses were produced according to the method described by Shakeel-Ur-Rehman *et al.* [45] by using either 100% chymosin or 100% *Munida* enzymes as coagulant [46].

Briefly, the freeze-dried *Munida* extract was re-suspended in 10 mM sodium phosphate buffer before use. After seven days, the freeze-dried extract entirely retained its activity with respect to the initial activity. Each preparation, diluted with water to 300 μL to have equal milk-clotting activity, was added to 200 mL milk. Three miniature Cheddar-type cheeses were manufactured in two batches on the same day using each of the two coagulants.

Cheeses were ripened at 8 °C and collected for analysis after 2, 6 and 12 weeks. Samples were taken, grated and frozen at −20 °C until analysis. The efficacy of the extracts in the manufacture of Cheddar mini-cheeses was determined by assessing their proteolytic ability over time in comparison with cheese made with chymosin. The *Munida* extracts showed high proteolytic activity on caseins in miniature Cheddar-type cheese. Strong β-casein activity was observed in the first 2 weeks of ripening, as detected by urea-PAGE of the pH 4.6-insoluble fraction of cheeses. Breakdown products obtained from αS1-casein were qualitatively different from the ones obtained using chymosin as coagulant. Patterns of proteolysis were also obtained by reverse-phase high-performance liquid chromatography and MALDI-ToF mass spectrometry. In general, the products of proteolysis were more complex in cheeses made using the *Munida* extracts than in cheese made with chymosin as coagulant. Statistical analysis of the results clearly discriminated between the cheeses on the basis of the coagulant used.

### 6.2. Ewe Mini-Cheeses

**Figure 3:**
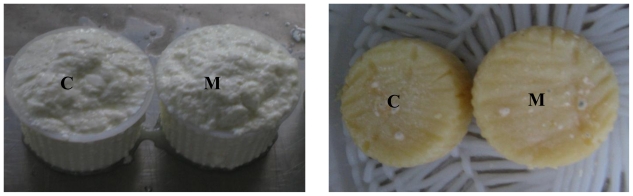
Mini pecorino-cheeses. Freshly made cheeses (left) and ripened 60 days cheeses (right). **C**: control; **M**: *Munida* pecorino.

Miniature (45 g) Pecorino-type cheeses were manufactured using the enzyme extracts obtained from the hepatopancreas of *Munida*. Briefly, heat-treated ewe’s milk (10 L) was cooled to 40 °C and termophilic lactic concentrated (CHOOZIT™ DVI, Danisco) was added as starter. After 30 min, a lamb rennet was added (2 mL per 10 L) and the milk was divided in two parts: the first was used as a control, whereas lyophilized *Munida* extract (700 U) was added to the second. The lyophilized extract used for the ewe-mini-cheese retained 85% of the initial activity after 4 months. Twelve mini-cheeses of about 85 g were obtained. 3% NaCl was added and the cheeses were stored at 8–12 °C and 85–90% relative humidity for 60 days (Figure 3). Analysis of the proteolytic pattern of casein fraction, performed by MALDI ToF mass spectrometry, showed the differences between the control and the *Munida* mini-cheeses and demonstrated that the *Munida* enzymes are capable of degrading caseins in an original manner.

## 7. Conclusions

In recent years, several new milk-clotting enzymes have been investigated as alternatives to calf rennet. They include recombinant chymosins produced using *E. coli*, *Kluyveromyces lactis*, or mammalian cells as hosts [47], *Rhizomucor miehei*, *Cryphonectria parasitica* [48], recombinant lamb chymosin [49], bovine pepsin, fungal proteinases from *Aspergillus niger* [50], *Rhizomucor miehei* [51], *Aspergillus Awamori* [52], and *Trichoderma reesei* [53], yeast proteinases from *Saccharomyces cerevisiae* [54] and *Candida Tropicalis* [55] and proteinases extracted from plants such as *Cynara cardunculus* [56–59], *Cynara humilis* [60], *Papilionoida* spp. [61], *Solanum dobium* [62] and *Centaurea calcitrapa* [63–65]. Instead, only a few digestive enzymes from marine species have been considered for their chymosin-like characteristics as potential substitutes for rennet, for their chymosin-like characteristics [14–18]. More recently, it has been reported that an acidic protease produced by the marine yeast strain *M. reukaufii* W6b possess milk-clotting activity [66].

However, rennet substitutes as the cardoon extracts often have a much greater level of non-specific proteolytic activity. This may lead to an extensive degradation of milk proteins and breakdown of the protein network, affect the texture of cheese and cause a reduction in yield and flavor development in cheese. Furthermore, the acceleration of cheese ripening, due to the higher proteolytic activity of the rennet substitutes, may result in increased bitterness in cheese. Among the enzymes with a possible application in the industry, those extracted from sea organisms certainly represent an economic benefit, since they come from fish catch usually thrown back into the sea. In particular, their use in food science represents a new possibility for upgrading fish waste and low value fish species to food with a high nutritional value. The advantages of marine enzymes to the food industry include the potential development of mild enzymatic methods in alternative to mechanical or chemical treatments, which may damage the product and lower its recovery rate. The property of the digestive enzymes from marine organisms to maintain their activity at low temperature might be very useful in food processing in order to avoid bacterial contamination and unwanted chemical reactions [13]. Thus, the use of marine enzymes in food technology is becoming a promising application for development of new processes and new products.

Analysis of the proteolytic pattern of the casein fraction performed by MALDI ToF mass spectrometry showed that the *Munida* enzymes are capable of degrading caseins in an original manner. The hydrolytic activities on α-casein and β-casein were indeed clearly different when compared with those of the commercial rennet. The *Munida* enzymes showed a higher activity on β-casein when compared to α-casein. The high degree of hydrolysis on α-casein and β-casein and the moderate clotting activity found in the extracts of the *Munida* crustaceans suggest their use in the dairy industry both for milk clotting, as an alternative or in addition to calf rennet, and for the acceleration of cheese ripening, to lower the time and costs of storage and maturation of cheese.

In addition, the peptides obtained from all caseins by the *Munida* enzymes might have a specific impact on flavor and texture characteristics of cheese. In this regard, the extracts obtained from the hepatopancreas were found to degrade the chymosin-derived β-casein fragment f193–209, one of the peptides associated with bitterness in cheese, revealing their possible application in cheese technology to lower the unpleasant bitter flavour in some cheeses.

Taken together, the application in cheese biotechnology of the *Munida* enzymes in combination with different peptidases (starter) seems to be highly promising for the production of cheeses with new characteristics. Future studies will be oriented to the purification of the *Munida* enzymes extracted from the hepatopancreas and to the determination of the best ratio to be used with rennet.
